# Down syndrome with microgranular variant of acute promyelocytic leukemia in a child: a case report

**DOI:** 10.1186/1752-1947-1-147

**Published:** 2007-11-24

**Authors:** Deepali Jain, Tejinder Singh, Prerna Arora

**Affiliations:** 1 Department of Pathology, Maulana Azad Medical College, New Delhi, India

## Abstract

**Background:**

Acute promyelocytic leukemia (APL) accounts for less than 10% of pediatric AML. Cases of APL in Down syndrome (DS) have been described in the literature rarely and it is rarer still to find the microgranular variant (M3v) of APL in trisomy 21 patients.

**Case presentation:**

We present a case of a five-year-old female with Down syndrome diagnosed with acute promyelocytic leukemia (APL). She came to our hospital with bleeding manifestations. Blood and bone marrow examination revealed promyelocytes showing a few fine granules and occasional Auer rods. Based on this morphology and cytochemistry, a diagnosis of APL microgranular variant (M3v) was made.

**Conclusion:**

This case report emphasizes the importance of a high index of suspicion in the diagnosis of acute promyelocytic leukemia microgranular variant in Down syndrome.

## Introduction

Acute myeloid leukemia (AML) comprises approximately one-fifth of pediatric leukemias [1]; and acute promyelocytic leukemia (APL) accounts for less than 10% of pediatric AML [2]. Trisomy 21 is associated with a 15-fold increased risk of acute leukemia, with AML occurring three times more frequently than acute lymphoblastic leukemia (ALL) in the first 3 years of life. The most common subtype of AML in Down syndrome (DS) is acute megakaryoblastic leukemia [3]. Though cases of APL in DS have been described in the medical literature rarely, it is rarer still to find the microgranular variant (M3v) of APL in trisomy 21 patients [4]. Herein, we describe a case of a five year old child with DS who presented with bleeding and was diagnosed as APL (M3v).

## Case presentation

**Figure 1:**
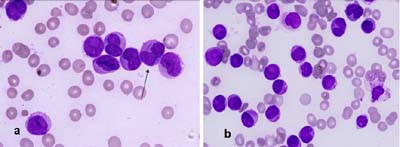
Photomicrographs of peripheral blood smear (a) and bone marrow (b) to show neoplastic promyelocytes and blasts with few fine granules and an occasional Auer rod (arrow in a), Wright – Giemsa ×1000.

**Figure 2:**
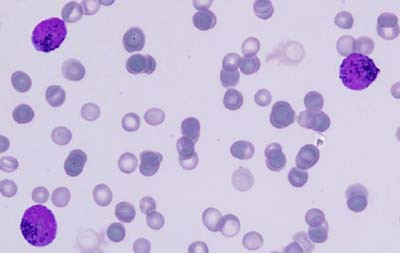
Myeloperoxidase stain shows strongly positive blasts and promyelocytes, ×1000.

**Figure 3:**
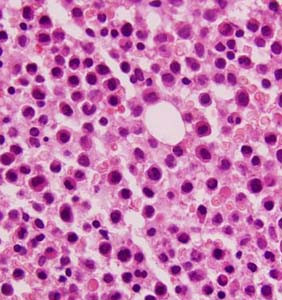
Photomicrograph of bone marrow biopsy showed hypercellular marrow infiltrated by sheets of blasts and promyelocytes and marked depression of normal haematopoiesis, H&E ×600.

A five-year-old female child, a known case of DS, presented with a history of gum bleeding, tarry stools and hematemesis of five days duration. On physical examination, she was pale and had petechiae, bleeding gums and hepatomegaly. There was an enlarged submandibular lymph node. Her coagulogram studies were unremarkable. Serum fibrinogen levels were within normal limits (2.0 g/l); and tests for fibrin degradation products and D-dimer were also negative. Hemogram findings were as follows: Hb 5 g/dl; WBC 80 × 10^3^/l; and platelets 12 × 10^9^/l. The peripheral smear demonstrated an increased number of WBCs, neoplastic promyelocytes and occasional blasts (90%) with high nuclear-cytoplasmic ratio and conspicuous nucleoli. These promyelocytes had markedly lobulated and invaginated nuclei. The cytoplasm of the cells contained no clearly recognizable granules but showed occasional Auer rods (Fig 1a). The red cells showed mild anisocytosis with a few macrocytes. A bone marrow aspirate and biopsy was performed with the following differential: 70% promyelocytes which had a morphology similar to those seen in the peripheral blood; 23% blasts; 5% myelocytes; and 2% lymphocytes. Normal hematopoiesis was suppressed (Fig 1b). The promyelocytes were strongly positive for myeloperoxidase (MPO) stain (Fig 2). Periodic acid Schiff's stain was predominantly negative except for fine granular positivity in an occasional promyelocyte. Unfortunately, conventional cytogenetic analysis could not be performed in this case due to unavailability of fresh blood or marrow sample after the diagnosis, as the patient left against medical advice. However, immunophenotypically these cells were strongly positive for anti MPO stain. A trephine biopsy showed hypercellular marrow infiltrated by sheets of blasts and promyelocytes and a marked depression of normal hematopoiesis (Fig 3). Based on the morphology and cytochemical analysis, a diagnosis of AML-M3v was made. All-trans retinoic acid (ATRA) with chemotherapy was prescribed. However, the patient left against medical advice. Therefore, the final outcome could not be ascertained.

## Discussion

APL is a subtype of AML in which abnormal promyelocytes predominate. APL accounts for approximately 5–8% of AML [5]. It differs from other subtypes of AML in that the patients are often younger. Our case was a 5 year old female with DS. Most patients of APL present with a pancytopenia rather than an elevated white cell count, however high WBC count has been seen in AML M3v. Our index patient had hyperleucocytosis at the time of presentation. AML M3v accounts for approximately 1/3 of APL cases. It most often presents in pediatric patients. The cytomorphology of AML M3v blasts is obviously different from AML M3 blasts, these cells have a non- or hypogranular cytoplasm or contain fine dust-like cytoplasmic azurophil granules that may not be apparent by light microscopy. Furthermore, M3v blasts show a typical bilobed, multilobed or reniform nuclear configuration. This latter morphologic phenotype, together with missing granulation, has often resulted in the misleading diagnosis of acute monocytic or myelo-monocytic leukemia. These cells show very strong MPO reaction, as was also seen in the case under discussion. Both AML M3 and M3v reveal translocation (15;17) (q22;q12) on cytogenetic analysis. Unfortunately cytogenetic analysis could not be done in the present patient. Immunophenotypically, they express myeloid phenotype [6]. Although 90% of children with AML have no known risk factors, a number of predisposing constitutional disorders have been found in the remaining 10% of children. Multiple studies have established the incidence of leukemia in DS patients, to be 10- to 20-fold higher than that in the general population. The proportion of ALL and AML in patients with DS is similar to non-DS leukemia patients matched for age. AML in DS is usually of the FAB M7 sub-type [3]. Only a few cases of APL associated with trisomy 21 have been published in the English literature. Singal et al [7] in 1987 reported a case of a 64-year-old male diagnosed to have APL with trisomy 21. Spell et al [4] in 2002 described M3-V in trisomy 21 patient in a 26-year-old Hispanic man. Recently, Kurkijan et al [8] published a case of DS with AML M3 encountered in an adult. Ours is the fourth reported case of APL in DS, and possibly the second case of AML-M3 v associated with DS. To the best of the authors' knowledge no case of APL in DS in pediatric age group has been described so far. It is important to report the variant form of APL, for therapy and prognosis of the patient. Results from gene profiling suggest that the two morphological subtypes of APL (M3 and M3v) are clearly separable. The present case adds a new case of pediatric AML M3v associated with trisomy 21 to the medical literature.

## Conclusion

This case report emphasizes the importance of a high index of suspicion in the diagnosis of acute promyelocytic leukemia microgranular variant in Down syndrome

## Competing interests

The author(s) declare that they have no competing interests.

## Authors' contributions

DJ wrote the case outline and performed the literature review. TS assisted in reviewing the slides. PA provided images. All authors read and approved the final manuscript.

## Consent

Written consent was obtained from the patient's relative for publication of this case study.
